# Primary colonic lymphoma as an uncommon cause of large bowel obstruction: a case report

**DOI:** 10.1097/RC9.0000000000001081

**Published:** 2026-10-06

**Authors:** Konstantina Kostara, Brandon Velazquez, Samantha Walkow, Tiffany Battagliola, Zbigniew Moszczynski

**Affiliations:** a Department of Surgery, Hudson Regional Health, Bayonne, NJ, USA; b Rowan-Virtua School of Osteopathic Medicine, Stratford, NJ, USA

**Keywords:** case report, colorectal cancer, diffuse large B-cell lymphoma, extranodal non-Hodgkin lymphoma, large bowel obstruction, primary colonic lymphoma

## Abstract

**Introduction and importance:**

Primary colonic lymphoma (PCL) is a rare extranodal manifestation of non-Hodgkin lymphoma, accounting for a small fraction of colorectal malignancies. Due to its low incidence and nonspecific clinical and radiological features, PCL is frequently misdiagnosed as colonic adenocarcinoma. Optimal management remains poorly defined, and current treatment strategies rely on retrospective data and expert consensus.

**Case presentation:**

We report the case of a 69-year-old woman who presented with right lower quadrant abdominal pain and was found to have a near-obstructing ascending colon mass with extensive regional and retroperitoneal lymphadenopathy on computed tomography. Colonoscopic biopsies were nondiagnostic despite a malignant-appearing lesion. Given impending obstruction, the patient underwent an open right hemicolectomy with primary anastomosis. Histopathologic and immunohistochemical analysis of the surgical specimen revealed diffuse large B-cell lymphoma (DLBCL). Postoperative staging with positron emission tomography demonstrated fluorodeoxyglucose-avid nodal disease. The patient was treated with adjuvant immunochemotherapy and radiotherapy, achieving a complete metabolic response on follow-up imaging.

**Clinical discussion:**

This case highlights the diagnostic challenges of PCL and emphasizes the importance of surgical resection in symptomatic patients for both definitive diagnosis and management. DLBCL is the most common PCL subtype and responds favorably to multimodal therapy, including rituximab, cyclophosphamide, hydroxydaunorubicin, oncovin, prednisone–based chemotherapy. Positron-emission tomography–computed tomography plays a critical role in staging and assessment of treatment response.

**Conclusion:**

PCL should be considered in the differential diagnosis of colonic masses, particularly when biopsy results are inconclusive. A multidisciplinary, multimodal treatment approach can lead to favorable outcomes. Further prospective studies are needed to establish standardized management guidelines.

## Introduction

Primary colonic lymphoma (PCL) is a rare lymphoid neoplasm originating in the colonic wall and must be distinguished from secondary colonic involvement by lymphomas of other primary sites^[^1,2^]^. While the gastrointestinal tract is the most common extranodal site of non-Hodgkin lymphoma (NHL), accounting for 30–40% of extranodal cases, the colon is the least frequently involved segment^[^3–5^]^. PCL represents only 0.2–1.2% of colorectal malignancies^[^4,6^]^ and poses diagnostic and therapeutic challenges due to its low incidence and nonspecific presentation^[^7,8^]^.

HIGHLIGHTS

- Primary colonic lymphoma is a rare cause of large-bowel obstruction.
- Clinical and endoscopic findings often mimic colorectal adenocarcinoma.
- Surgical resection may be required for diagnosis and symptom control.
- Multimodal therapy can achieve favorable oncologic outcomes.

The incidence of PCL has increased from 1.0 per million in 1973 to 3.5 per million in 2014^[^4^]^. It typically presents in the sixth to seventh decade of life, with a male predominance of approximately 2 to 1^[^4,9^]^. The cecum is the most common site, likely due to abundant lymphoid tissue in the ileocecal region^[^1,7^]^.

Although the etiology of PCL remains unclear, known risk factors mirror those of nodal NHL. Immunosuppression is a recognized risk factor^[^7,10,11^]^. Inflammatory bowel disease, particularly ulcerative colitis, has also been implicated, although the increased risk may be related to long-term immunomodulator therapy rather than inflammation alone^[^5,12^]^.

The majority of PCLs are B-cell NHLs^[^1,2,5,6,13^]^. Diffuse large B-cell lymphoma (DLBCL) is the most common histologic subtype, accounting for 40–81% of cases^[^6,13^]^ and is typically more aggressive than other B-cell variants. Accurate histopathologic classification is essential due to differences in clinical behavior and treatment response^[^5,7^]^.

Symptoms are nonspecific and depend on the location and size of the lesion. Abdominal pain is the most common symptom; other symptoms include weight loss, anorexia, changes in bowel habits, and gastrointestinal bleeding^[^1,6,13–17^]^. Systemic “B” symptoms are uncommon, contributing to delayed diagnosis and advanced-stage presentation^[^1,2,14,15^]^.

Contrast-enhanced computed tomography (CT) is the primary imaging modality for assessing tumor extent and nodal involvement, but it cannot reliably distinguish lymphoma from adenocarcinoma^[^3,15,17^]^. Definitive diagnosis requires histologic and immunohistochemical evaluation of adequate tissue samples^[^5^]^. Colonoscopy allows direct visualization and biopsy^[^8,12,17^]^. Because diagnosis can be challenging, multi-point biopsy should be performed^[^3^]^. Fine-needle aspiration is generally inadequate for determining the specific lymphoma subtype^[^5^]^.

The 2014 Lugano classification is the current standard for staging NHL and incorporates PET-CT imaging for prognostic and therapeutic planning^[^18–21^]^. This system differentiates localized (Stage I–II) from disseminated disease (Stage III–IV), which is critical for treatment decisions^[^8^]^.

Management of PCL requires a multidisciplinary approach. Given the rarity of PCL, current guidelines are limited by a lack of high-level evidence^[^21^]^.

Surgical resection is indicated for complications such as obstruction, perforation, or bleeding and provides diagnostic tissue^[^13,17^]^. The surgical strategy for localized disease involves resection to remove all gross disease^[^21^]^. Resection has shown survival benefits in patients with early presentation, right-sided lesions, and diffuse large B-cell histology^[^4,22,23^]^.

DLBCL is highly sensitive to multiagent immunochemotherapy^[^5^]^. The standard treatment is the cyclophosphamide, doxorubicin, vincristine, and prednisone (CHOP) regimen^[^18^]^. The addition of rituximab (R-CHOP) significantly improves progression-free disease interval and overall survival rates for CD20-positive B-cell lymphomas^[^23^]^. Surgical resection followed by R-CHOP has demonstrated 3-year disease-free rates exceeding 80% and, in certain studies, has been shown to outperform immunochemotherapy alone^[^5^]^.

Radiotherapy (RT) is generally used only in select cases^[^6,13^]^. RT may be employed for local control following incomplete surgical resection or as an aggressive local treatment for early-stage localized follicular lymphoma. However, its application is limited due to the potential for acute and chronic bowel toxicity^[^5,13,14^]^.

Although the condition has historically been associated with a poor prognosis, 5-year survival rates now range from 27 to 58%^[^13,15,18^]^, with a median overall survival of 95 months^[^4^]^.

This case report has been reported in line with the SCARE checklist^[^24^]^.

## Case presentation

A 69-year-old woman with a history of hypertension was evaluated by the General Surgery service on March 13 for a right colon mass identified during an emergency department visit for right lower quadrant pain on February 22. CT imaging during that visit demonstrated a poorly defined right colon mass with mural thickening of the cecum and ascending colon, loss of fat planes between adjacent small-bowel loops, right iliac adenopathy measuring 2.7 × 4.1 cm, extensive retroperitoneal lymphadenopathy, and enlarged para-aortic nodes (Fig. 1).

**Figure 1.**
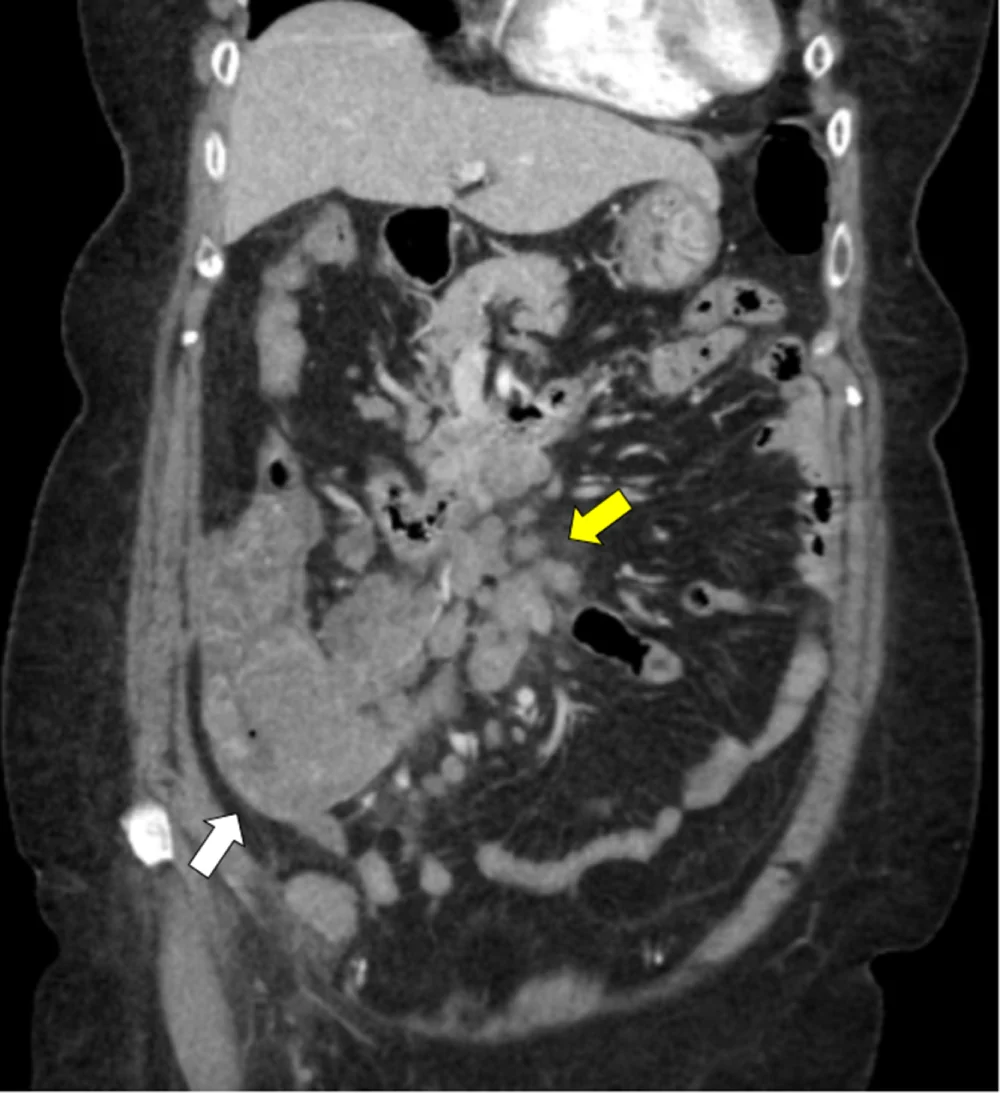
CT scan demonstrates mural thickening of the cecum and ascending colon (white arrow), right iliac adenopathy, and extensive retroperitoneal and para-aortic lymphadenopathy (yellow arrow).

The patient reported a family history of colon cancer in a sister who was diagnosed at age 49. She denied any other family members with cancer. She denied any allergies, smoking, alcohol use, or illicit substance use. She denied constitutional symptoms. Laboratory studies, including a complete blood count, comprehensive metabolic panel, and coagulogram, were consistent with adequate nutritional status and were notable only for mild anemia, with a hemoglobin level of 11.0 g/dL.

Colonoscopy was performed on 27 February, which revealed a fungating, partially obstructing mass in the ascending colon approximately 60 cm from the anal verge, which could not be traversed. The lesion was biopsied and tattooed. An incidental 8-mm sessile sigmoid polyp was removed (Fig. 2). Pathology showed chronic inflammation and ulceration without malignancy, and repeat biopsy was recommended given the uncertainty of the results. The incidental sigmoid polyp was an adenoma with high-grade dysplasia.

**Figure 2.**
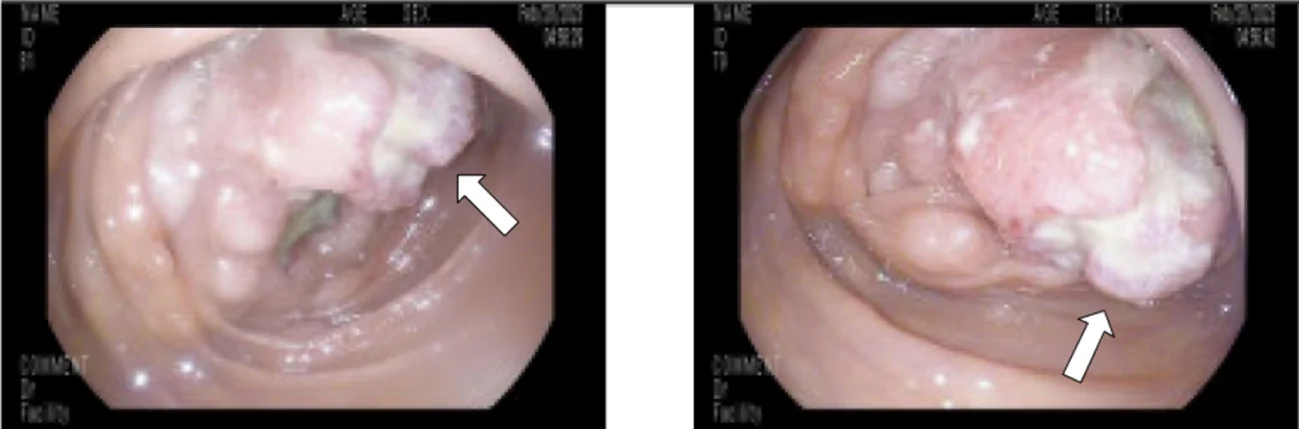
Colonoscopy reveals a fungating, partially obstructing mass in the ascending colon (white arrows).

Given the progressive symptoms of near-complete bowel obstruction, radiographic findings concerning for malignancy, and nondiagnostic endoscopic biopsies, the decision was made to proceed with an exploratory laparotomy and open right hemicolectomy with primary ileocolic anastomosis on 13 March. Through a midline laparotomy, exploration revealed a large right lower quadrant mass enveloping the terminal ileum and cecum with bulky regional mesenteric lymphadenopathy. The right colon was extensively mobilized to the hepatic flexure with meticulous adhesiolysis, and the right ureter was identified and preserved throughout the dissection. An en bloc resection of the terminal ileum, cecum, ascending colon, and proximal transverse colon with associated mesentery was performed, including high ligation of the ileocolic vessels. Intraoperative inspection demonstrated multiple enlarged lymph nodes in the peripancreatic, para-aortic, and pericaval regions; frozen section analysis of representative nodes demonstrated no evidence of malignancy. Intestinal continuity was restored with a primary ileocolic anastomosis, which was reinforced with an omental patch. The abdominal cavity was copiously irrigated; a Blake drain was placed adjacent to the anastomosis; and the abdomen was closed in standard fashion. The patient tolerated the procedure well and was extubated prior to transfer to the recovery unit.

Although intraoperative frozen-section analysis of representative enlarged lymph nodes showed no evidence of malignancy, permanent histopathologic examination demonstrated DLBCL in the separately submitted mesenteric and peripancreatic lymph nodes. The right hemicolectomy specimen showed DLBCL involving the cecum, proximal ascending colon, ileocecal valve, and appendix, with lymphoma identified in 24 of 24 lymph nodes. The ileal and colonic resection margins were negative. The neoplastic cells expressed CD20, PAX5, CD45, BCL2, and BCL6, with weak c-MYC expression, and were negative for CD10, MUM1, and cyclin D1. The Ki-67 proliferation index was markedly elevated at 80–90%.

Fluorescence in situ hybridization confirmed a BCL6 rearrangement in 84% of analyzed cells. Rearrangements involving MYC and BCL2 were not detected, although additional copies of MYC, BCL2, and BCL6 were observed, consistent with a neoplastic polyploid clone. Therefore, the findings did not meet molecular criteria for a MYC-associated double-hit or triple-hit lymphoma.

Mechanical venous thromboembolism prophylaxis with compression stockings was initiated immediately after surgery, with pharmacologic prophylaxis planned for postoperative day 1. However, before anticoagulant prophylaxis could be initiated, the patient developed a lower-extremity deep vein thrombosis on postoperative day 1 and was treated with therapeutic anticoagulation for 5 months following discharge. The patient was discharged on postoperative day 5 and was briefly readmitted 4 days later for self-limited hematochezia.

PET-CT a month after surgery demonstrated fluorodeoxyglucose (FDG)-avid intra-abdominal and pelvic lymphadenopathy (Fig. 3). Based on the extent of abdominal nodal involvement, the disease was classified as Lugano stage II2, and a chemotherapy port was subsequently placed. The patient received immunochemotherapy and RT, although the treatment sequence and RT indication, field, dose, fractionation, and dates could not be verified from the available records. Follow-up PET-CT at 5 months showed no FDG uptake, indicating a complete metabolic response. At the last follow-up available for this report (10 months), she remained under oncologic surveillance (Table 1).

**Figure 3.**
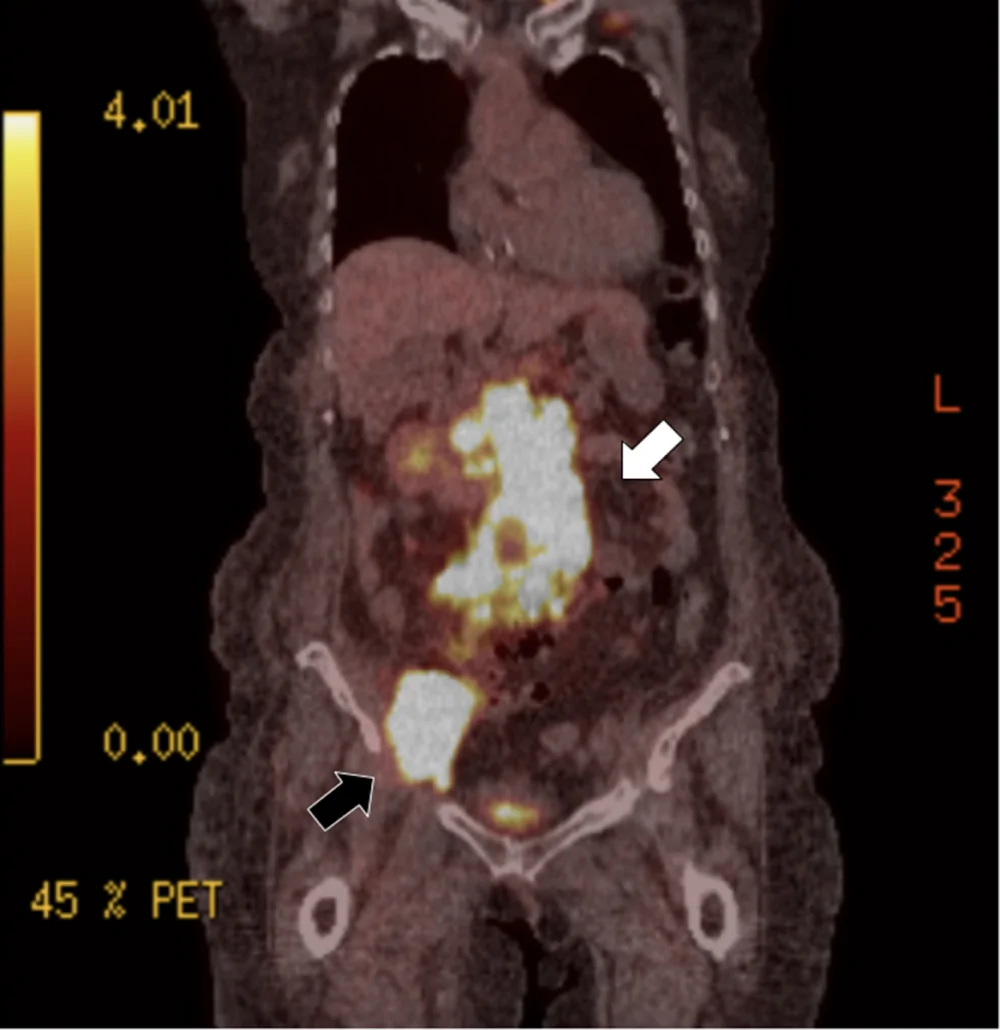
PET-CT demonstrating FDG-avid intra-abdominal and right pelvic lymphadenopathy (white and black arrows, respectively).

**Table 1:** Timeline of the clinical course.

Date or interval	Clinical event
22 February 2023	Presented to the emergency department with right-lower-quadrant abdominal pain. CT showed cecal and ascending-colon mural thickening with loss of adjacent fat planes, right iliac adenopathy, and extensive retroperitoneal and para-aortic lymphadenopathy.
27 February 2023	Colonoscopy identified a fungating, partially obstructing ascending-colon mass that could not be traversed. Biopsies showed inflammation and ulceration without malignancy; an incidental sigmoid adenoma with high-grade dysplasia was also identified.
13 March 2023	Underwent exploratory laparotomy and open right hemicolectomy with primary ileocolic anastomosis for near-complete obstruction. Representative lymph-node frozen sections were negative for malignancy.
14 March 2023	Developed a lower-extremity deep-vein thrombosis and began therapeutic anticoagulation, which was continued for 5 months.
18 March 2023	Discharged after initial postoperative recovery.
22 March 2023	Briefly readmitted with hematochezia, which resolved without further intervention.
31 March–3 April 2023	Permanent histopathology established diffuse large B-cell lymphoma involving the cecum, proximal ascending colon, ileocecal valve, appendix, and all 24 resected lymph nodes; mesenteric and peripancreatic nodes were also involved, and resection margins were negative. Ki-67 was 80–90%. FISH detected a BCL6 rearrangement (84% of cells) but no MYC or BCL2 rearrangement.
1-month follow-up	Staging PET-CT showed FDG-avid intra-abdominal and pelvic lymphadenopathy. The disease was classified as gastrointestinal Lugano stage II_2_, and a chemotherapy port was placed.
Subsequent months	The patient received immunochemotherapy and radiotherapy.
5-month follow-up	PET-CT demonstrated a complete metabolic response.
10-month follow-up	The patient remained under clinical and imaging surveillance.

CT, computed tomography; DLBCL, diffuse large B-cell lymphoma; DVT, deep-vein thrombosis; FDG, fluorodeoxyglucose; FISH, fluorescence in situ hybridization; PET-CT, positron-emission tomography–computed tomography.

## Discussion

PCL presents with nonspecific symptoms and endoscopic findings that frequently overlap with those of colorectal adenocarcinoma, including circumferential masses, ulceration, luminal narrowing, and obstruction^[^2,5,12^]^. Other considerations include inflammatory bowel disease, infectious colitis, and, less commonly, gastrointestinal stromal tumors or metastatic disease. In our patient, the obstructing colonic mass and imaging findings initially raised strong suspicion for colorectal adenocarcinoma. However, despite the grossly malignant appearance of the lesion, colonoscopic biopsies demonstrated only acute and chronic inflammation without evidence of malignancy. This likely reflects the predominantly submucosal growth pattern of PCL, which may limit the diagnostic yield of superficial mucosal biopsies. Although image-guided biopsy of enlarged regional or retroperitoneal lymph nodes may be considered in selected patients with suspected lymphoma, our patient’s impending bowel obstruction and persistent diagnostic uncertainty favored upfront surgical resection, which provided both definitive histopathologic diagnosis and therapeutic relief of the obstruction^[^2,5,7,11,12^]^.

Several definitions have been proposed to distinguish primary gastrointestinal lymphoma from secondary gastrointestinal involvement by systemic lymphoma. The classic Dawson criteria require the absence of peripheral and mediastinal lymphadenopathy, a normal peripheral white blood cell count, predominance of the intestinal lesion, lymph-node involvement limited to the regional drainage basin, and absence of hepatic or splenic involvement^[^25^]^. Our patient did not strictly satisfy the regional-node component because peripancreatic, para-aortic, and pelvic lymphadenopathy was present. We therefore applied the broader operational definition proposed by Lewin *et al*, in which a lymphoma is considered primary gastrointestinal when the gastrointestinal tract contains the predominant clinical lesion or is responsible for the presenting symptoms^[^26^]^. In this patient, the dominant bulky cecal and ascending-colon lesion, presentation with impending bowel obstruction, absence of peripheral or mediastinal lymphadenopathy, absence of hepatic or splenic involvement, and lack of a previously identified nodal lymphoma supported classification as PCL rather than systemic lymphoma with incidental secondary colonic involvement. The distant intra-abdominal nodal disease was consequently classified as stage II₂ under the gastrointestinal Lugano staging system^[^18–21^]^.

Multicenter trials are needed to reach a consensus on operative treatment. Additionally, assessing the long-term efficacy of modern treatment regimens, including R-CHOP, requires extended follow-up and large-scale studies^[^3^]^. Finally, advancing the molecular understanding of PCL through research in genetics, tumor biology, and immunohistochemistry will be essential for improving management and tailoring therapies more effectively^[^8^]^.

Molecular testing by FISH confirmed a BCL6 rearrangement in 84% of analyzed cells, whereas MYC and BCL2 rearrangements were not detected. Although BCL2 and weak c-MYC expression were observed by immunohistochemistry, these findings should be distinguished from gene rearrangements and did not establish molecular double-hit or triple-hit disease. The tumor demonstrated a high Ki-67 proliferation index of 80–90%. Despite these high-risk pathological features and extensive abdominal nodal involvement, the patient achieved an early complete metabolic response following combined-modality therapy^[^3,14,15^]^.

Although the role of RT in PCL remains controversial, selected patients may benefit from consolidative treatment following systemic chemotherapy, particularly those with localized residual disease, bulky tumors, or incomplete metabolic response^[^6,13^]^. Current evidence supporting RT is limited and derives primarily from retrospective studies, with treatment decisions individualized within a multidisciplinary setting.

Limitations include the 10-month follow-up, lack of broader genomic profiling and quantitative MYC and BCL2 protein-expression data, and unavailable RT records, which precluded determination of its indication and treatment parameters.

This case highlights several important clinical lessons. First, PCL should remain in the differential diagnosis of an obstructing colonic mass, even when imaging and endoscopic findings strongly suggest colorectal adenocarcinoma. Second, negative endoscopic biopsies do not exclude malignancy, as the predominantly submucosal growth pattern of PCL may limit the diagnostic yield of superficial mucosal sampling. Finally, in patients with impending bowel obstruction or persistent diagnostic uncertainty, surgical resection may serve both therapeutic and diagnostic purposes, allowing definitive histopathologic diagnosis and guiding subsequent oncologic management.

## Conclusion

PCL is a rare malignancy that often mimics colorectal adenocarcinoma and presents at an advanced stage due to nonspecific symptoms. Accurate diagnosis requires a high index of suspicion and histopathologic confirmation. Management typically involves surgical resection for symptomatic disease, followed by systemic chemotherapy, with RT reserved for selected cases. Given the absence of standardized treatment guidelines, current management relies on retrospective data and expert consensus. Prospective multicenter studies are needed to define optimal therapeutic strategies and improve long-term outcomes.

## Data Availability

The data supporting the findings of this case report are not publicly available due to patient privacy and confidentiality considerations. Relevant de-identified data are contained within the article.
